# Definition of the Ethical Values and Ethics Codes for Turkish Midwifery: A Focused Group Study in Kocaeli

**DOI:** 10.5812/nms.11222

**Published:** 2013-09-15

**Authors:** Ayla Berkiten Ergin, Müesser Özcan, Nermin Ersoy, Zeynep Acar

**Affiliations:** 1 Department of Midwifery, Kocaeli High Health School, University of Kocaeli, Kocaeli, Turkey; 2 Department of Nursing, Muğla High Health School, University of Muğla Sıtkı Koçman, Muğla, Turkey; 3 Department of History of the Medicine and Medical Ethics, Medical Faculty, University of Kocaeli, Kocaeli, Turkey

**Keywords:** Midwifery, Codes of ethics, Social values, Social characteristics

## Abstract

**Background:**

The independent roles of midwives have not been properly defined, and midwifery ethical values and moral codes proper to Turkish culture have not been developed. The absence of legal regulations concerning midwifery has negatively affected midwifery in the process of professionalization.

**Objectives:**

The purpose of this study was to identify the professional values of midwifery in Turkey.

**Materials and Methods:**

A focus group was created with the participation of nine midwives working at two state hospitals and a university hospital that provide birth service for women in Kocaeli, which is the most important industrial city in Turkey. The opinions of the midwives on the characteristics that a good midwife should possess and the professional values that a good midwife should observe were collected via in-depth interviews. The interviews were recorded. A total of three meetings were held with the participants. Finally, the notes taken by the reporter during these interviews were rearranged, and the recordings were transcribed by the researchers.

**Results:**

The characteristics suggested by the participants were classified into three categories: professional, personal, and interpersonal. Professional competence, capacity to properly inform interested parties, trustworthiness, respect for individuals and human dignity, and empathy were the most commonly named characteristics. As for the professional values of midwifery, professional competence, trustworthiness, responsibility, maximum benefit, and protection of privacy were the most often identified. Midwives also reported that most of the difficulties they faced in the exercise of daily tasks concerned protecting the privacy of their patients as well as the integrity and prestige of the profession, achieving the maximum benefit and least harm for patients, and providing a just and equal service.

**Conclusions:**

The professional values were mentioned by participant midwives were similar to the values proposed by international professional organizations. But there were some differences perhaps due to cultural differences.

## 1. Background

Midwifery is a professional discipline which combines science, art, and strong ethical values. Midwifery, which is one of the oldest professions for women in the world, has always been respected and highly regarded in the Turkish culture, and the women facilitating labor were perceived as goddesses (1, 2). Midwifery was considered to be a strictly female profession, and the profession was passed down from one generation to the next in a system of mentorship/apprenticeship, especially from mothers to their daughters. Considered practitioners of a well-respected profession, midwives during the Ottoman rule were ordinary members of society who nevertheless achieved an excellent reputation and great fortune (3). Despite the historical importance of midwifery, the social status of midwifery in contemporary Turkey is disconcerting. The independent roles of midwives have not been properly defined, no midwifery ethical values and moral codes proper to Turkish culture have been developed, and the absence of legal regulations concerning midwifery has negatively affected midwifery in the process of professionalization. However, midwifery education started as early as in 1842, and midwifery was the first profession which Turkish women were able to achieve through education (3-5). Midwifery practice is based on professional and personal values formed by the physiological realities of pregnancy and birth and surrounded by cultural values affecting women and babies (6-8). Taylor et al. (2008) defined value as “a personal belief about worth that acts as a standard to guide one’s behavior” (9). A system of values involves an organization arranging its components in order of importance to act as a code of behavior. Positioned at the intersection of science and art and grounded in moral values, midwifery as a professional healthcare discipline might be one of the rare professions which has continuously observed a set of fundamental values from the days of its first practitioners (10, 11). Because of the nature of their profession, midwives provide service to both women and their newborns throughout the labor process, which is laden with various cultural values. They do all of this according to their own personal and professional values (6-8, 12). Therefore, many studies that focused on determining and updating the ethical code and values of midwifery were initiated in the US and many European countries in the 1970s (13). We planned this long overdue study to investigate the fundamental values of midwifery that help attain the prime goal of this profession. 

## 2. Objectives

The object of this study was to identify the ethical values of midwifery proper to Turkey in light of local cultural values, which impact both individual and professional values.

## 3. Materials and Methods

This project was prepared in cooperation with the Kocaeli University Department of Medical History and Ethics and the Kocaeli Health Vocational School Department of Midwifery to determine the professional values of midwifery. The project consisted of two stages. In the first stage, the opinions of midwives on the values of their profession were collected using in-depth interviews. This article discusses the methods employed and findings obtained in this first stage.

Turkish midwives work at Family Health Centers, Society Health Centers, hospitals in the delivery rooms, obstetrics clinics, gynecology clinics, newborn clinics, and family planning polyclinics general. This study was conducted in Kocaeli. With its population of 1.5 million Kocaeli is an important industrial city bordering the Black Sea and Marmara Sea. A great number of people have been migrated from different regions of Turkey to Kocaeli. So as a city it was a good example to represent the social structure of the whole Turkey. Two to three midwives were selected from each of three hospitals in downtown Kocaeli that had a birth clinic facility. These midwives work in obstetrics, delivery and healthcare units. The sampling was based on voluntary criteria. These midwives represented all other volunteering midwives working at the same institution and composed the study group of this study. The sampling was finished on 12.12.2010. They were informed in advance of meeting times and locations.

Three different hospitals which accommodated a delivery room and obstetrics clinic and were located in central Kocaeli constituted the universe of the study. From each hospital, administrators selected three or four midwives among the volunteers with at least five years of experience to represent their colleagues for a total of nine midwives who formed the study group. The study was conducted with 12 individuals including one moderator, two specialists and one reporter. Focus group discussion is defined in the literature as a research methodology involving a conversation on a predefined topic supervised by an expert on the topic in question to reveal the viewpoints and attitudes of a group of 6-12 participants guided by questions prepared by the researcher beforehand, whereby the outputs of the participants are outlined. The present study was therefore designed with respect to the general characteristics of focus group discussion methodology. The participants were seated in a circular arrangement such that each participant could see the others in a room designed to allow group work. The study was initiated with twelve individuals, i.e. nine midwives, one supervisor (moderator), two specialist academicians and one reporter. Care was taken to avoid that the specialists other than the moderator expressed their viewpoints within the group. The moderator was a professor in ethics and an experienced academician in the topic of values. The moderator was supported by two specialists. The first had a doctorate degree on this topic of ethics and was an experienced academician. The other had a doctorate degree on obstetrics and gynaecology nursing who was also experienced in nursing. It was the responsibility of the specialists to ensure that the participants relaxed before the study and that the physical order was maintained. Three meetings were held with the participant midwives (the first on 18.01-2011, the second on 15.02.2011, and the final discussion on 18.03.2011). The major strength on the present study was that the group behaved naturally owing to the fact that the meetings were held in a completely social atmosphere, the group members exchanged their opinions in a natural environment and they experienced increasing satisfaction. The participants made a circle in a room, especially prepared to accommodate a group study in such a way that everybody could see each other. The study started with the participation of a total of twelve individuals: nine midwives, one moderator, two educational experts, and a reporter. The experts were discouraged from voicing their opinions in the group with the exception of the moderator. The topics and subtopics to be discussed in the meetings were meticulously prepared and listed in advance. All questions were determined before the meetings and were subjected to a pretest. All participants were given a specific number, and all comments were recorded. It was ensured that the notes taken during meetings were orderly and of good quality. A special form was prepared to record the minutes of the meetings on which positive and negative statements were grouped into two columns by the reporter. Also all meetings were recorded with a voice recorder with the permission of the participants. The topic was introduced by the moderator at the beginning of each meeting, and the discussions were followed by questions about interpretation. At the end of each meeting, the subjects covered in the discussions were concisely summarized, and everybody was allowed to reiterate their arguments to prevent any misunderstandings. The meetings lasted one and a half hours without any breaks. A total of three meetings were held with the participants. The second meeting took place two weeks after the first one, and the third meeting was held ten days after the second one. In the first meeting, the questions “What characteristics should a good midwife possess, and which values should be prioritized in the practice of midwifery?” were asked to guide the discussion. In the second interview, the participants were asked about what should be performed to observe ethical values in the practice of clinical midwifery and about the situations that challenge them the most in applying these ethical values in the clinics. They were also asked about the ways these challenges could be overcome. In the last interview, researchers shared their categorized responses with the participants. Inaccuracies were revised, and missing points were added at the suggestions of the participants. Finally, the notes taken by the reporter during these interviews were rearranged, and the recordings were transcribed by the researchers. Researchers read a copy of the text and came together to discuss the material until they reached agreement on primary and secondary themes. The study was converted to a report, including some of the participants’ responses. The comments and the opinions of the participants were then synthesized.

This study was conducted with the approval of the Kocaeli Clinical Research Ethics committee (KKAEK 6/11) and with the permission of the Kocaeli Provincial Directorate of Health. Necessary information was given about the study to the participants and their informed consents were taken. Also researchers have informed the participants about their rights to leave the study at any time they want to.

## 4. Results

The participants had a mean age of 28 years and a mean professional experience of seven years. Five of nine participants graduated from high school, and four of them had university-level training. The first part of the collected data was about the opinions of the participants on the characteristics that a good midwife should possess, while the second part was about the preferences of the participants concerning the values of midwifery. The third part was about the challenges faced by midwives in putting values into practice and their proposals for overcoming these challenges.

### 4.1. Characteristics That a Good Midwife Should Possess

The qualities named by the participants were classified into the following three categories: professional, personal, and interpersonal relationships and communication (Table 1). Some of the qualities in each of these categories were named by most of the participants and some by all of them. For example, professional competence, respect for individuals, and informing interested parties properly were among the professional characteristics most commonly named, as were trustworthiness among the personal characteristics and the capacity to empathize. Among the characteristics that the participants believed that a good midwife should possess, professional competence, capacity to inform interested parties properly, trustworthiness, respect for individuals, and the capacity to empathize were the most commonly named characteristics. Below is a selection from the statements of the participants that underscore the above mentioned qualities (the letters in parentheses at the end of the statements are the codes of the participants composed of the initial letters of their names and surnames): 

A midwife should be professionally competent; “Although philanthropy, cheerfulness and intimacy are very important characteristics, the most important characteristic that a midwife should possess is professional competence. Midwifery cannot be performed without sufficient professional knowledge and skills” (D.Ö).

“I am both angry with and ashamed of my incompetent colleagues who have difficulties facilitating the labor process and doing the follow-ups” (H.D).

“Professional knowledge and skills are a must. Someone without them cannot be rightly called a midwife” (Y.D).

“The professional competence of a midwife is above all other values. Professional competence should be prioritized over others” (F.A.Ç).

“When I first started working as a midwife, I got really upset when a man, who was waiting for his wife’s labor, left the hospital telling me that I looked very inexperienced and that he could not leave the two persons that he loved the most to an inexperienced person like me. However, because of what I have witnessed over the years, I now think that he was right” (A.T).

A midwife should be able to inform interested parties properly; “I think a midwife should take into account that the parties she is addressing might not have a high level of education or they might have problems comprehending what is said because of the situation they are in and should thus present clear and understandable information tailored for to the needs of individuals” (T.Y.).

“Midwives should present information in such a way that any misunderstanding on the part of the pregnant woman and her family is reduced to a minimum, and thus she should use very simple sentences in explaining the situation”(F.A.Ç).

“Women might refuse a procedure even like placing a catheter if they are not informed properly. When a midwife explains a procedure clearly enough, women would get rid of their fears and submit themselves to the midwife. Otherwise, women would be very tense and resist the procedure” (H.D).

“Women in Turkey cannot quite comprehend what is said to them. Especially when they come here to deliver, their level of comprehension goes even lower because of the fear of the pain they expect to experience. Therefore, I explain all information over and over again, especially if the woman to deliver has a low level of education” (D.Ö).

A midwife should be trustworthy; “Midwives witness the most difficult and private moments of women. Women should be able to trust them” (Y.D).

“Women and their families should have confidence in midwife throughout the whole labor process” (F.A.Ç).

“The words and actions of midwives should be trustworthy, and midwives should be able to strike others as a trustworthy individual” (H.D).

A midwife should respect individuals; “Some patients are discriminated against; when I witness such incidents, I warn my colleagues against discrimination. Every individual deserves respect” (F.A.Ç).

“I am ashamed of midwives who insult women who scream a lot during the labor process. I am ashamed of being a member of the same professional community as them” (T.Y).

A midwife should be able to empathize; “After all, midwives are also women, and they can very well understand what a woman goes through” (D.Ö).

“I could not empathize very much with pregnant women before I gave birth to my own child. Now I remember my own experiences in every labor process, and I should confess that I am more considerate of women now. Therefore, midwives should be able to empathize with pregnant women” (A.T).

“I have never given birth myself, but I think empathizing is a matter of professionalism. One does not have to give birth before he or she can empathize with pregnant women” (T.Y).

The view that “Giving birth is a moment that mothers would remember for rest of their lives” is why midwives should turn these special moments into memories that would be remembered positively with their own characteristics and the values that they observe” (T.G.), which was expressed by one of the participants but shared by all of them, explains why it is necessary to be a good midwife.

The second category of data in this study was about the opinions of the midwives on their preferences concerning midwifery values.

**Table 1.** Characteristics That a Good Midwife Should Possess

Qualities
Professional Characteristics
Midwives should care about the well-being of mother and child
Midwives should regard the health of mothers as their first priority
Midwives should respect individuals and human dignity^*^
Midwives should commit to midwifery as a profession
Midwives should seek to provide the maximum benefit and do minimum harm
Midwives should treat everyone equally
Midwives should pay attention to and protect privacy
Midwives should improve themselves and their professional knowledge
Midwives should be competent^*^
Midwives should be able to foresee possible damage and risks and be able to take measures to prevent them
Midwives should be able to keep a secret (be a confidant)
Midwives should be responsible
Midwives should be able to inform interested parties properly^*^
**Personal Characteristics**
Midwives should be trustworthy^*^
Midwives should protect their own prestige
Midwives should be resolute (patient and tenacious)
Midwives should have confidence in themselves
Midwives should be humble
Midwives should be able to communicate effectively
Midwives should be patient
Midwives should have inner peace
Midwives should be altruistic
Midwives should be philanthropic
**Interpersonal Relationships and Communication**
Midwives should be able to empathize with others^*^
Midwives should be cheerful
Midwives should be persuasive
Midwives should have good communication skills
Midwives should be intimate
Midwives should be sympathetic

^*^ The most frequently named characteristics

### 4.2. Preferences Concerning the Values of Midwifery

The participants named 16 values as the fundamental ethical values of the profession of midwifery (Table 2). The participants put professional competence at the top of their list of professional values, just as they did for the characteristics of a good midwife. Similarly, trustworthiness and protection of privacy were also considered to be among the values with the highest priority. 

One of the participants explained her position on trustworthiness as follows: “We are also women. Women should be able to trust us. They should be able to feel that we care for them. They should be able to believe that we are going to protect their privacy and be able to notice that we are concerned with them (A.T.). Another participant said, “Midwives should work without embarrassing women and make sure that women feel good” (TY).

One of the midwives explained why she thought midwives should have aesthetic values: “I would not like to be remembered as a bad person in a woman’s memory of her difficult times. This is why I always try to be empathetic, polite, and thoughtful even when I am very tired, sad or nervous” (T.Y.).

The last part of the data concerned the challenges faced by midwives in putting these values into practice and their proposals for solutions.

**Table 2.** 

	Professional Values^*^
**1**	Competence
**2**	Trustworthiness
**3**	Responsibility
**4**	Maximum benefit
**5**	Respect for privacy
**6**	Equality
**7**	Preventing pain
**8**	Courage
**9**	Effective communication
**10**	Empathy
**11**	Accuracy
**12**	Respect for human dignity and individuals
**13**	Updating professional knowledge
**14**	Loyalty to the profession
**15**	Responsibility
**16**	Sincerity

^*^ Listed in the order of preference frequency

### 4.3. Challenges Faced by Midwives in Their Practices and Their Proposals for Solutions

The values that midwives had difficulty observing in their practices were found to be similar to the characteristics that they thought a good midwife should possess and the fundamental values of midwifery. The values that midwives had difficulty observing or putting into practice were among the list of characteristics that a good midwife was supposed to have. For example, respecting privacy was not only one of the values that midwives had difficulty observing but also one of the characteristics that the participants thought a good midwife should possess and one of the listed professional values. Similarly, seeking the maximum benefit for women in professional practice was also included in the list of necessary qualities for a good midwife (seeking the maximum benefit and minimum harm for women) and also in the list of professional values (seeking the maximum benefit). The challenges faced by midwives in treating women justly and equally without discrimination caused the participants to include “not to discriminate” both among the necessary qualities of a good midwife and the list of professional values to be observed. The challenges faced by midwives in protecting the integrity and prestige of their profession were also transferred to the list of professional values as loyalty to midwifery. In addition to these challenges and ethical dilemmas, some participants offered solutions. These solutions for coping with the challenges faced by midwives in putting professional values into practice also established the groundwork for a possible ethical code for the profession. The challenges faced by midwives and the solutions they offered are presented in their own words (Table 3). 

**Table 3.** Values that Midwives Have Difficulty Observing and Proposals for Solutions

Challenge: Protecting Privacy
Suggestions
Physical and ethic measures should be taken both on individual and institutional levels concerning the conducting of medical interventions and midwifery services.
Women should be informed of the process in such a way that they can understand so they are willing to give their consent.
Women should be given the opportunity to practice their right to decide or to refuse as autonomous individuals, and they should even be encouraged to practice this right.
Health personnel should assume responsibility for satisfying the health-related needs of women, fetuses or babies who are not in a position to protect their own rights.
**Challenge: Protecting the Integrity and the Prestige of the Profession**
**Suggestions**
Midwives should not seek personal interests in the services they provide and should not establish any relationship with individuals or institutions on the basis of a personal interest.
Midwives should be in a respectful, constructive, effective, and intimate dialogue with their colleagues and with their patients to increase the quality of healthcare.
Trustworthiness and accuracy should be ensured in all practices and patient records as required by the obligation of honesty. In addition, midwives should avoid misinforming pregnant women and should report incorrect practices.
Midwives should adopt attitudes and behaviors that reflect the dignity and the prestige of their profession.
Midwives should get involved in scientific studies and contribute to the development of midwifery, strengthening of professional organizations, and education of colleagues. They should improve their professional skills and knowledge.
Midwives should listen to their consciences and use them as a guide for actions and self-evaluation.
**Challenge:Maximum Benefit, Minimum Harm**
**Suggestions**
Seeking the maximum benefit for the women and babies should be a priority in all practices.
Midwives should prevent women and their babies from being subjected to inattentive or inexperienced practitioners and prevent any possible related harm.
Midwives should ensure that women are able to use existing medical facilities safely and prevent any possible related harm.
Experienced difficulty: Treating Everybody Justly and Equally Without any Discrimination
Suggestions
Midwifery services and resources should be allocated justly according to the needs of individuals.
Every pregnant woman, unborn child, mother and baby should be regarded as an individual and receive equal treatment.
Midwives should believe that everyone has equal rights, and they should provide service to everyone without discrimination.

## 5. Discussion

These focus group interviews were conducted with an eye toward determining local midwifery values in Turkey, on the characteristics that a good midwife should possess and on the values of midwifery. The opinions of the midwives who participated were found to be similar to the generally accepted philosophy and duties of midwifery (13, 14). Many opinions held by the participants, namely that a good midwife should care about the well-being of women and babies, that she should regard the health of mothers as her top priority, that she should respect individuals and their rights, and that she should be professionally competent, were found to be similar to the values established by professional midwifery organizations. Many national and international organizations such as the International Confederation of Midwives (ICM), the Midwives Alliance of North America (MANA), and the Association of Turkish Midwives declare that it is a midwife’s duty to protect the health of mothers and babies and to avoid inflicting any harm on them. They even define midwives within the context of community service as health activists who work in cooperation with women to protect their rights and the rights of their families (13, 15, 16). The participants’ view that a good midwife should possess such characteristics as trustworthiness, strong communication skills, and the capacity to maintain a high level of empathy, sincerity, cheerfulness, and philanthropy coincides with the principles established by the American College of Nurse Midwives (ACNM). ACNM similarly emphasizes that a good midwife should respect human rights, human dignity, their own self-worth, and their own professional integrity and should possess such qualities as being reassuring, honest, truthful, merciful, caring and respectful (13, 17). As for the values of midwifery, our study group prioritized professional competence, professional responsibility, and seeking the maximum benefit for the mother and the child as the most important. This result is comparable to the professional values proposed by international professional organizations. According to the ACNM, ensuring that midwives are competent in their knowledge and skill, and that their competence is continuous is a necessity (13, 18). The participants, who witness women’s most intimate moments, considered the protection of privacy to be one of their highest priorities. This reinforces the values proposed by international professional organizations such as MANA and ACNM as well as helping to define the nature of the profession (16, 17). However, there was a difference between the lists in the ranking of this value, perhaps due to cultural differences (13, 14, 18). Although these results suggest that midwives observe the values that are highlighted in the national midwifery anthem and the national midwifery education such as professional competence, respect, protecting lives, alleviating pain and suffering, compassion, objectivity (justice), secrecy, honesty, and respect for privacy, it is unsettling that they were having difficulty practicing these values. Midwives in the focus group reported that they were having difficulty protecting the privacy and dignity of women, respecting them as individuals, protecting the integrity and prestige of the profession, achieving the maximum benefit for the woman and the child, and providing service justly and equally in their daily practice. The participants thus demanded that institutional measures be taken, that rules be introduced, and that compliance with these rules be monitored to alleviate these problems. The views of the focus group on the characteristics of a good midwife and on the ethical values of midwifery resemble the ethical codes and values declared by such international institutions as ICM, ACN, and MANA. However, there were some minor differences. Showing that cultural values might impact professional values, these results indicate the importance of determining the professional values of midwifery and professional ethical codes in accordance with the local culture and circumstances of a given country. This study was therefore conducted as a preliminary study to determine the ethical codes and values in Turkey, and the Association of Turkish Midwives was subsequently contacted to initiate a cooperative nationwide study in light of the findings of this study.

Occasional distractions experienced within the group and sporadic deviations from the topic were the major limitations of the present study. Some participants were more willing to speak while others were more reluctant, and the moderator’s attempts to establish a balance represented another challenge. Planning the study according to the working hours of the participants and creating a homogenous group were other difficulties experienced during the study.
